# GPU-friendly data structures for real time simulation

**DOI:** 10.1186/s40323-021-00192-7

**Published:** 2021-03-27

**Authors:** Vincent Magnoux, Benoît Ozell

**Affiliations:** Department of Computer Engineering and Software Engineering, Polytechnique Montréal, Chemin de Polytechnique, Montréal, Canada

**Keywords:** Surgery simulation, GPU computing, Cutting simulation, Physically-based simulation

## Abstract

Simulators for virtual surgery training need to perform complex calculations very quickly to provide realistic haptic and visual interactions with a user. The complexity is further increased by the addition of cuts to virtual organs, such as would be needed for performing tumor resection. A common method for achieving large performance improvements is to make use of the graphics hardware (GPU) available on most general-use computers. Programming GPUs requires data structures that are more rigid than on conventional processors (CPU), making that data more difficult to update. We propose a new method for structuring graph data, which is commonly used for physically based simulation of soft tissue during surgery, and deformable objects in general. Our method aligns all nodes of the graph in memory, independently from the number of edges they contain, allowing for local modifications that do not affect the rest of the structure. Our method also groups memory transfers so as to avoid updating the entire graph every time a small cut is introduced in a simulated organ. We implemented our data structure as part of a simulator based on a meshless method. Our tests show that the new GPU implementation, making use of the new graph structure, achieves a 10 times improvement in computation times compared to the previous CPU implementation. The grouping of data transfers into batches allows for a 80–90% reduction in the amount of data transferred for each graph update, but accounts only for a small improvement in performance. The data structure itself is simple to implement and allows simulating increasingly complex models that can be cut at interactive rates.

## Introduction

Despite decades of progress, realistic real-time surgery simulation remains computationally challenging. Calculating the deformation and behavior of organs according to physical models is complex and needs to be done very fast to produce a virtual environment that is responsive to user actions, especially when haptic feedback is desired. The calculations become even more demanding when cutting operations need to be performed, such as the resection of a tumor, while the organ and surrounding tissue are being deformed. The behavior of soft tissue is itself non-trivial to simulate, but adding cuts and other topology-changing operations means that many acceleration structures that allow increased performance can no longer be precomputed.

One approach to improve the resolution and realism of simulations consists in making better use of the constantly increasing capacity of common computer hardware, such as multi-core processors (CPU) and graphics processors (GPU).

In general, multi-threaded CPUs allow performing multiple different tasks in parallel, usually 4 to 16, or subdivide a task and execute its parts concurrently. In contrast, a GPU may achieve a high level of parallelism, on the order of thousands of concurrent threads, as long as they all perform the same computation.

Physically based simulation lends itself relatively well to GPU processing when the problem is reduced to solving a sparse set of linear equations at every step. However, when introducing topology changes in the simulated object, the coupling between these linear equations changes and the precomputed data that allows solving them quickly must be updated.

In order to minimize the cost of such updates caused by a cutting operation, we propose a new data structure that avoids any sort of reallocation of GPU memory and that reduces the amount of data that needs to be copied after many small changes are made to it.

### Background

Before discussing GPUs specifically, we first summarize the main methods used for performing physical computations for surgery simulation. The simplest method is with a mass-spring system (MSS), where a set of points—the masses, or particles—are connected through springs, which introduce axial forces between the points when they are stretched or compressed [1]. While MSS are easy to implement, it is difficult to choose the right spring stiffness parameters that will accurately simulate the behavior of soft tissue. Finite element methods (FEM) provide a more realistic model of deformable bodies by solving the continuum elasticity equations over a domain divided into elements [2]. They are also referred to as mesh-based methods, since the elements form a mesh. In contrast to FEM, meshless methods solve these equations with a more diffuse discretization of space, where the “elements” are less geometrically defined and may overlap each other [3]. They are also called particle-based methods. Position-based dynamics (PBD) is another successful approach that may offer the accuracy of FEM or the flexibility of MSS and meshless methods, depending on how the particles are connected together through constraints [4].

These methods ultimately all depend on solving a system of equations. Assembling and solving that system are often the most computationally intensive aspects of the simulation, and therefore the ones that must be targeted for GPU acceleration to achieve the best performance improvement.

Two aspects of GPUs that make them notoriously difficult to use efficiently will be discussed in this paper:

- Operations must be structured in a way that allows hundreds of threads to simultaneously read from memory and perform the same set of computations on the data. This is referred to as the single instruction, multiple threads (SIMT) execution model [5].
- On a GPU, the memory space is different and *not* shared with that of the CPU. Data must be transferred between the two sets of memory whenever the host or GPU make a change that must be read by the other.

Early implementations of solvers were made using a graphics API [6, 7]. It however came with severe restrictions on how the data is structured—using textures rather than arrays—and on the available precision—only 24-bit floating points could be used. The development of general purpose GPU computing (GPGPU) platforms such as CUDA [5] allowed for much more flexibility and complexity in the kinds of solvers that could be implemented.

In the most general sense, a system solver gathers data about a system, such as forces or constraints, and based on this information, determines in what state the system will next be, usually referring to the positions of various nodes forming an object. In our case, the system consists of deformable organs, a surgical tool and any other simulation element that may interact with them.

The simplest solvers are usually explicit ones, which only require to evaluate nodal forces and accelerations at or before the current time, allowing to find the velocities and positions at the end of the time step. They have been used on the GPU in surgery simulation with MSS [8, 9], FEM [10–12] and meshless methods [13]. Since it requires a low amount of computations, it can relatively easily be combined with a haptic device [12, 14], which requires a high refresh rate, or with expensive computations like cutting, other physical phenomena like melting [13], or direct volume rendering [15].

The main downside to explicit solvers is their stability. Even if they are very fast, they require a certain small time step size that depends on the size of the smallest element in the simulated object and on its rigidity.

Implicit solvers provide a much higher stability, at the cost of having to solve a non-linear system, which requires more computations. They however allow arbitrarily long time steps. Surgery simulators using that integration scheme usually solve the system using a matrix-free, iterative solver, which requires less memory than a direct one while allowing for topology changes between frames. These solvers have been used to simulate non-linear behavior [16], cutting a tesselated surface embedded in a meshless model [17], in combination with a compliance method for resolving interactions [18] or with constraints to model permanent deformation and cutting [19].

Position-based dynamics offers a way to combine many aspects of a simulation such as deformation, phase changes, liquids et collision detection and response into a single method. It uses a two-phase process—also called prediction-correction scheme—to first move particles freely in time, based on their current speed, then correct their position directly based on a set of constraints. The constraints are solved using a highly parallelizable Gauss-Seidel method. Organ deformation has been simulated using shape-matching constraints [20] or energy constraints [21]. The latter offer a more physically realistic behavior and provide a simple way to cut the object, by removing and adding constraints.

Other methods have been used to simulate deformations on the GPU, like a multigrid iterative solver [22], a direct static solver [23], or an iterative static solver [24]. However, these methods introduce new levels of complexity or rigidity that make them more challenging to use for cutting simulation and, in the case of static solvers, prone to sudden reactions when a user interacts with the simulated objects.

### Contributions

In this paper, we propose a simple way to store the graph data describing the relationship between the nodes of an object that is both efficient to access on the GPU and easy to modify from the CPU and to update. We describe it as part of a simulator based on the Element Free Galerkin (EFG) method [3] using an implicit solver, but it is extendable to other methods such as FEM and may be of benefit when using other solvers.

## Method

We first describe the computations needed for determining how a simulated object moves and gets deformed before explaining how we structure the data to efficiently perform these computations on the GPU while allowing for topology changes. While the discussion focuses on an elasticity problem, our solution can be applied to other simulations that make use of a graph-like structure.

### Deformation

We wish to solve the continuum equations of elasticity for a dynamic system:

1 $$\begin{aligned} \rho \ddot{\varvec{u}} = \nabla \cdot \varvec{\sigma }+ \varvec{f}^{ext}\text {,} \end{aligned}$$

where $\rho$ represents mass density, $\varvec{u}$ the displacement field, $\varvec{\sigma }$ the stress and $\varvec{f}^{ext}$ any external forces. Note that since we use a linear elasticity model, $\varvec{\sigma }$ only depends on $\varvec{u}$. After discretization into a set of nodes and linearization, we obtain a system of equations

2 $$\begin{aligned} \varvec{M}\ddot{\varvec{u}} = \varvec{K}\varvec{u}+ \varvec{f}\text {,} \end{aligned}$$

with $\varvec{M}$ as the mass matrix, $\varvec{K}$ as the stiffness matrix, $\varvec{u}$ the vector of nodal displacements and $\varvec{f}$ the vector of external nodal forces.

For a surgery simulation with haptic interactions, stability is essential. We thus choose to use an implicit dynamic solver, which remains stable for large time steps. Additionally, unlike explicit solvers, the time step is not constrained by the smallest element size, which is difficult to control when arbitrary cutting is allowed. Following the method of [25], we obtain

3 $$\begin{aligned} \left( \varvec{M}- \Delta t^2 \varvec{K}\right) \Delta {\dot{\varvec{u}}} = \Delta t \left( \varvec{f}^{elastic}_0 + \varvec{f}^{ext}_0 \right) \text {,} \end{aligned}$$

where $\Delta t$ is the length of the time step, $\Delta {\dot{\varvec{u}}}$ the change in velocity during that time step – the quantity we are trying to determine – and $\varvec{f}_0 = \varvec{f}^{elastic}_0 + \varvec{f}^{ext}_0$ the total forces on the object nodes at the beginning of the time step. From the solution to eq. 3, we can compute the new nodal displacements as

4 $$\begin{aligned} \varvec{u}= \varvec{u}_0 + \Delta t ({\dot{\varvec{u}}}_0 + \Delta {\dot{\varvec{u}}}) \text {,} \end{aligned}$$

where $\varvec{u}_0$ and $\dot{\varvec{u}_0}$ are respectively the nodal displacement and velocities at the beginning of the time step.

The entries in $\varvec{K}$ are determined by the relationships between nodes, whether they are connected through elements in mesh-based methods or through their influence in meshless methods. Since we are constantly changing these connections by cutting, $\varvec{K}$ also changes constantly, making the use of a precomputed system matrix impossible. Additionally, because the computation of $\varvec{K}$ is expensive, we prefer to use a matrix-free method, such as conjugate gradient (CG), for solving the linear system. In that case, the main computation becomes the multiplication of $\varvec{K}$ with an arbitrary vector of nodal displacements (or corrections) at each iteration of the solver (see Algorithm 1).

As for the mass matrix term $\varvec{M}$, we lump the object’s mass on the nodes, resulting in a diagonal matrix. Its product with a vector can thus be reduced to an element-wise multiplication that can be added to the left-hand side of Eq. 3.
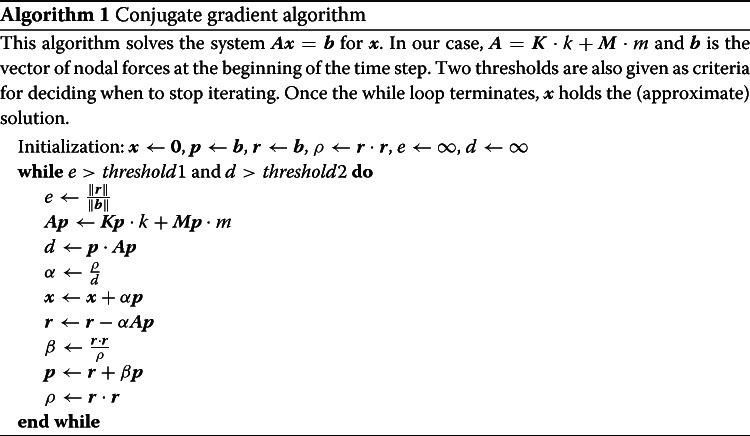


The computation of the matrix-vector product in Algorithm 1, in particular the $\varvec{K}\varvec{p}$ product, is driven by the structure that connects the nodes together. For example, with finite elements, the domain may be subdivided into tetrahedra, where each tetrahedron connects four nodes. With the EFG method, which we use in the present work, each cubic integration element combines a set of about eight nodes. However, the computation method is the same regardless of how the elements are formed. Algorithm 2 describes how we compute $\varvec{K}\varvec{p}$ by looping over the elements, compute their force density, and distribute it to their nodes.
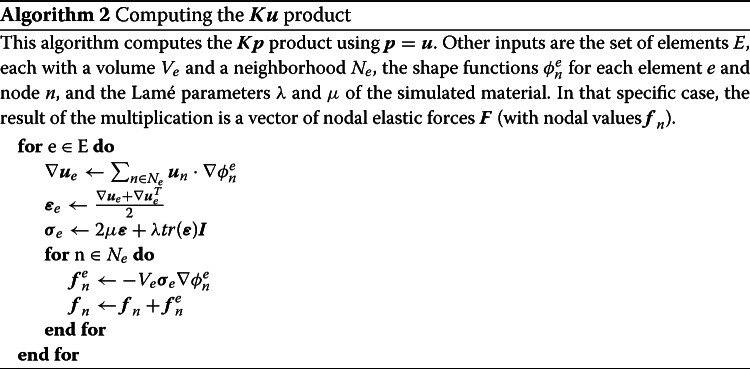


The rest of the conjugate gradient consists of relatively simple vector operations, such as additions and scalar products, which can easily be carried out on the GPU. As pointed out and implemented by [18], for each iteration, only two scalars need to be transmitted to the CPU to determine whether to continue iterating.

The operations presented in this section represent the most intensive part of the simulation and are thus the ones that need to be targeted for a GPU implementation. However, they depend on data structures that are modified every time a topology change occurs in the simulated model and must be implemented in a way that is not overly penalized by these changes.

### Changing connectivity data

We now describe how the element connectivity data is encoded so that it can be efficiently modified on the GPU. As a general rule for GPU computing (also for optimal cache access on the CPU), we want pieces of data that will be accessed together to be located close to each other in memory.

**Fig. 1:**
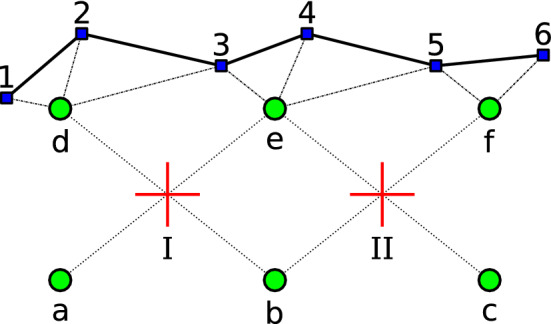
Connectivity arrays. Illustration of what the four large 2D data arrays needed for updating an object’s state represent. Red crosses are the center of elements, green circles the simulation nodes and blue square are surface vertices. The connectivity links elements to nodes, with shape function values and derivatives associated with each pair. The surface mapping links vertices to nodes, with a weight associated with each pair

We perform topology changes using the method presented in [26] and map the surface onto the physical model as in [27]. There are four sets of data, illustrated in Fig. 1, that are very large and need to be updated every frame:

1. The connectivity graph that assigns a set of nodes to each element; for example, element I would be $\left\{ i \big |i \in {a, b, d, e}\right\}$
2. The shape function value for each element-node pair; I = $\left\{ \phi _i^I \big |i \in {a, b, d, e} \right\}$
3. The shape function derivative for each element-node pair; I = $\left\{ \nabla \phi _i^I \big |i \in {a, b, d, e} \right\}$
4. The weights of the mapping between surface vertices and nodes; for example, vertex 3 would be $\left\{ w_i^3 \big |i \in {d, e} \right\}$.

They are represented in the diagram of Fig. 1. The connectivity between nodes and surface vertices actually uses the same graph as the connectivity between nodes and elements, and thus requires little additional data to update.

**Fig. 2:**
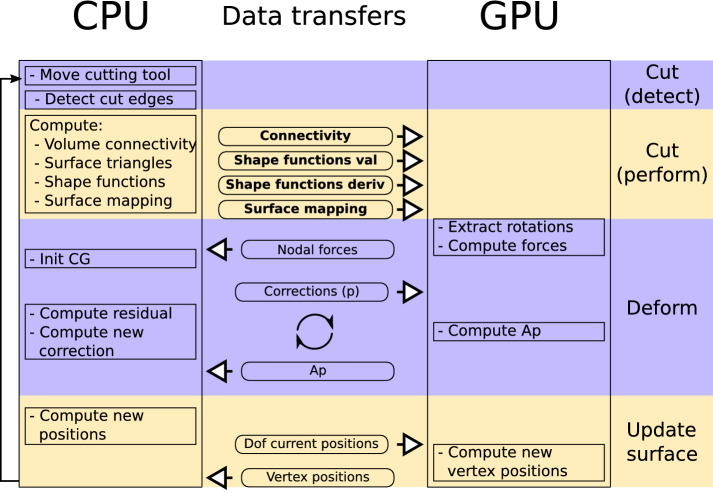
Execution flow. Execution steps and data flow of the main simulation loop highlighting which operations are performed on the CPU and on the GPU. Boldface data are the large 2D structures described in this paper

Figure 2 illustrates which operations of the simulation are performed on the CPU and which are performed on the GPU. It also shows what data need to be transferred for each of these operations. Names in bold are the 2D arrays discussed in this section.

Two considerations guided our choice of data structure for storing the connectivity and shape functions and mapping: avoid constantly reallocating memory on the GPU and group many small memory transfers into a larger batch—to avoid the relatively large latency associated with each transfer.

These four data sets may be viewed as two-dimensional arrays. For example, the element-node connectivity has a row for each element, containing the identifier of the nodes that are connected to that element. Unlike mesh-based methods, the number of nodes per element may vary, so the rows have uneven lengths.

A memory-efficient way to store such a 2D array, as done for example by [16], would be to have a linear array containing all rows contiguously, with a second array indicating where each row starts in the larger one, and a optionally a third one to store the length of each row. However, with such a structure, if a row increases in length from one frame to the next, all subsequent values in the larger array would need to be shifted. That would be almost equivalent to updating the entire data structure, in addition to having to reallocate memory for it. The cost of a new allocation as well as en entire copy would be noticeable in an application where fast update rates are required.

**Fig. 3:**
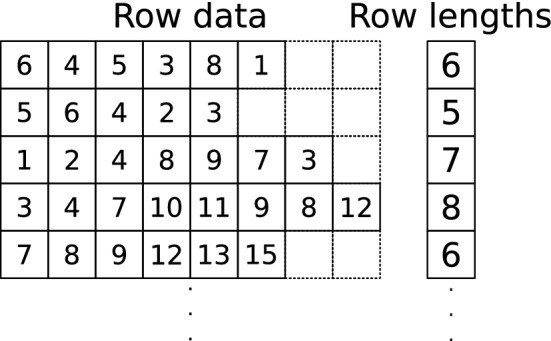
Custom data structure. Data structure used for major 2D arrays—connectivity data is illustrated here. In the first, large array, rows all occupy the same amount of memory, regardless of the number of elements in them. A secondary array contains the number of elements (length) in every row

To avoid having to reallocate memory, even after a change in size for some rows, our solution is to set a maximum row size and allocate a single block that is overall slightly larger than what is strictly needed. To keep track of the actual number of items in each row, we also add a vector that lists the current size of each row. This two-array structure is illustrated in Fig. 3. Since no elements are added during the simulation, the row size and data block arrays will never need to be resized or reallocated.

This structure is somewhat less flexible than the compact array, because the number of nodes attached to an element can never exceed the allocated maximum row size. However, the criterion that determines whether we need to add nodes in the neighborhood of an element is whether these nodes are coplanar (see [26] for details). The cases where this condition cannot be satisfied when choosing at least 8 neighbors are theoretically rare and will be discussed further in “Limitations” section.

During a single time step, only a small fraction of all elements will have a new set of neighbor nodes. Similarly for surface changes, only a relatively small number of vertices will need a new mapping onto the volume. To avoid copying the entire data structure and the overhead of many small memory transfers, we divide the data arrays into batches, which can be copied one at a time to GPU memory. The batches are all part of the same memory allocation, only memory transfers are affected by this division. This reduces the total amount of data to be copied every time step while keeping the number of memory transfers low.

**Fig. 4:**
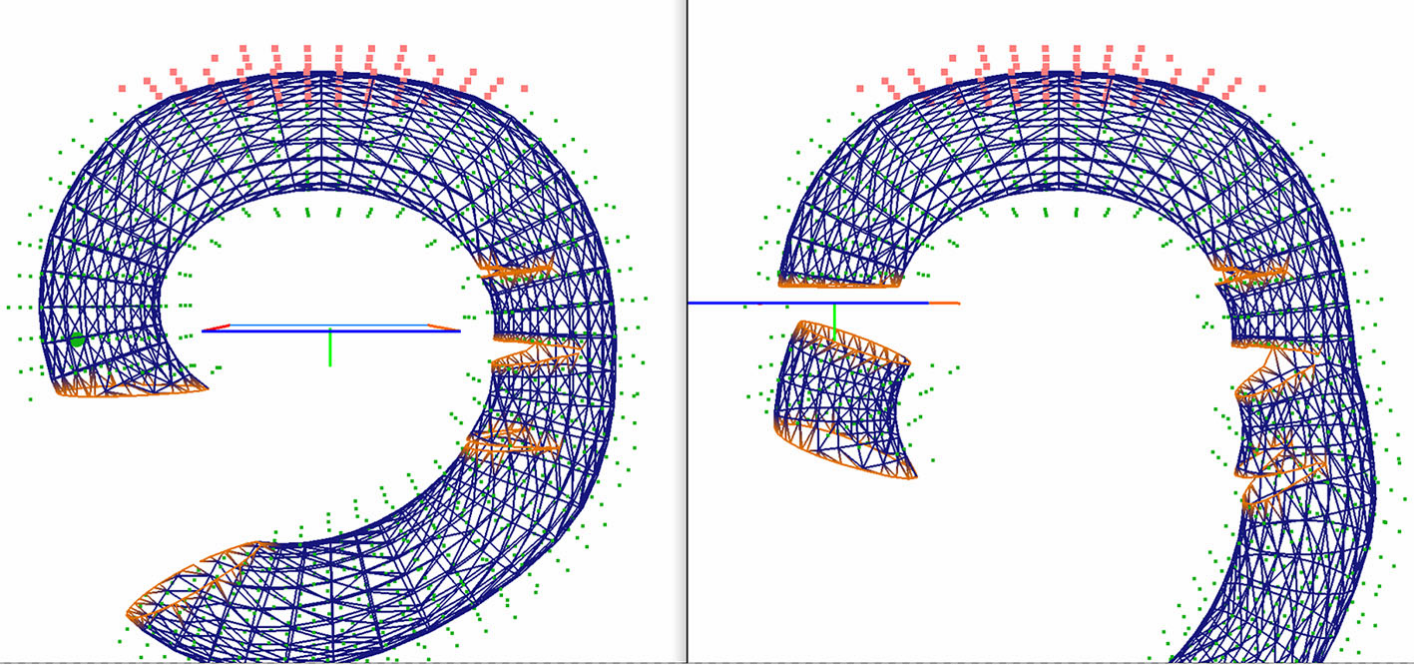
Snapshots of the test case. Snapshots of the torus used as a test model, with a low element and triangle count to better display the geometry

## Results and discussion

To examine whether the particular data structure described in “Method” section allows for an efficient simulation on the GPU and how the performance evolves in different situations, we have implemented the method described in [26] using CUDA and incorporating that structure. The model used to demonstrate cutting is a torus made of a varying number of elements (from 1.5k to 80 k) and surface mesh size (from 1.5 k to 50 k triangles). It was cut using a virtual tool made of several segments and animated as described in [26]. The tool followed a predetermined trajectory, and the torus would deform under gravity before, during and after the cutting procedure. Figure 4 and the accompanying videos demonstrate the execution of this test case with a low number of elements and surface triangles to show details of the geometry. All tests whose results are presented in this section were run on a 6-core Intel Core i5-9400F CPU and an Nvidia RTX 2060 GPU.

**Fig. 5:**
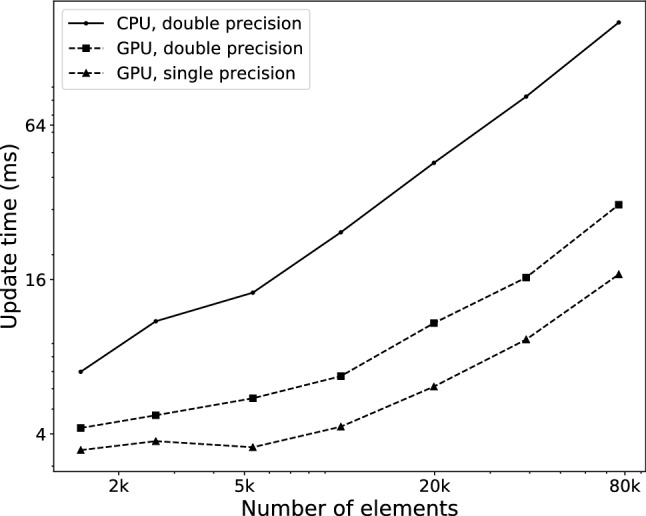
Update time per element. Total update time (in ms) for different configurations, with respect to number of elements in physical model

### Performance comparisons

We first compared the execution time of each simulation update—or time step—of the simulation when run on the CPU only and when using the GPU for computing deformation, in each case using both single and double precision floating-point arithmetic. Figure 5 displays the evolution of each update execution time with respect to the number of elements in the simulated model. The scene only contains a torus being partially cut at various volumetric resolutions, with a surface of approximately 50k triangles. The total time displayed includes every aspect of the simulation, which can be categorized into cut detection, cut application, deformation computation and surface position update, as in Fig. 2.

We can observe in Fig. 5 that the GPU version is faster at all problem sizes, and that the difference only grows larger as the number of elements increases. The relatively worse performance of the GPU version for smaller problem sizes is due to the fact that the 2k-core GPU itself is not used to its full capacity.

Using single precision arithmetic, the GPU version maintains a rate of 60 frames per second while cutting a model containing 80k elements, whereas the CPU version reaches that rate at about only 6k elements. When modeling an entire human brain or liver, these numbers translate to element sizes of approximately 2 mm and 6 mm respectively. The 60 frames per second criterion is used as the reference for which we can have a fully interactive simulation where the physics behavior update rate is the same as the visual update rate. This number may be relaxed somewhat depending on the requirements of the simulator regarding interactivity, user immersion or hardware capacity.

CPU performance is only shown for double precision computations, since it was slightly better than single precision performance. At larger sizes, the single-precision GPU version is approximately 45% faster than the double-precision version. This speedup could be improved slightly further by also using the GPU to detect which edges are cut, for both volume and surface. The cutting and surface mapping algorithm however does not lend itself well to a parallel implementation [27].

**Fig. 6:**
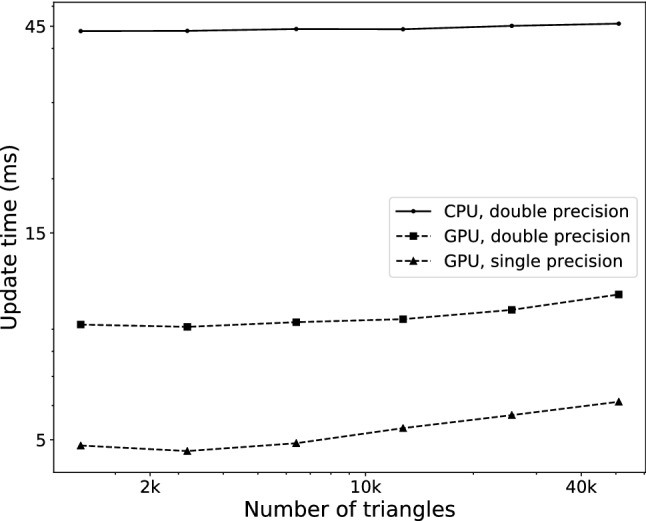
Update time per triangle. Total update time as a function of the number of triangles in the surface mesh. The volumetric model used for these tests has 20k elements, which was the approximate size limit to obtain an interactive update rate on the CPU

To determine how the size of the surface mesh affects the performance of the GPU implementation, we also examined how the total computation time evolves with an increasing surface mesh resolution. The results are shown in Fig. 6, using a physical model of 20k elements that allowed for interactive refresh rates on the CPU. It shows that the surface mesh size barely affects simulation time. This result indicates that the growth of simulation time related to surface operations (cut detection, cut application and position update) is much smaller than that of the time related to volume operations, which is taken mostly by deformation computation, and to a lesser extent by volume cutting.

**Fig. 7:**
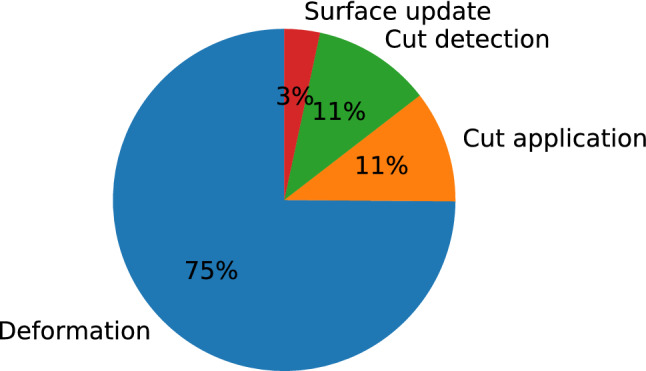
Total update proportions. Proportion of execution time taken by each major step of the main simulation loop, for the case using single precision floating points on the GPU, with 20k volume elements

Figure 7 displays the proportion of execution time taken by each major step of the main simulation loop. The deformation step, even when running on the GPU, takes the largest proportion with 75% of the update time. Magnoux et al. [26] showed that this proportion increases with the number of volume elements, but decreases slightly with a larger number of surface triangles.

**Fig. 8:**
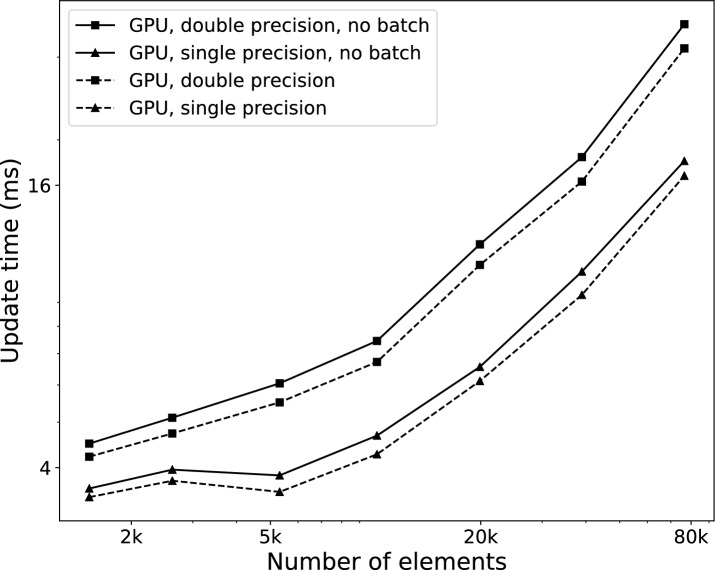
Update time with batches. Comparison of total update times for the GPU version of the simulation with and without splitting 2D arrays of data into batches, both in single and double precision

Figure 8 displays the running times of the same set of simulations as Fig. 5, for the GPU versions where the splitting of 2D arrays into batches was either activated or not. It shows a regular performance improvement of approximately 7% in single precision and 11% in double precision for all volumetric model sizes. The speed gain is however proportional to the size of the surface mesh (which was of 50k triangles in this test). This indicates that the savings engendered by splitting the 2D arrays are more important for the surface mapping data, which happen to form the largest of all arrays in almost all tested cases—the exception being very refined volumes displayed in very coarse surfaces. In our test cases, splitting the arrays into batches resulted in a reduction of 80 to 90% in the amount of data transferred during each simulation update.

**Fig. 9:**
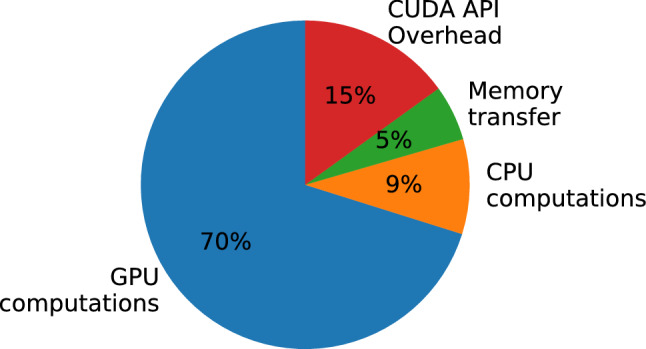
Deformation time proportions. Proportion of execution time taken by different aspects of the deformation step, using single precision. Computations take most of the time, mainly on the GPU, while memory transfer and CUDA overhead remain low

The better gains shown with double precision suggest that a more important proportion of the simulation is spent copying data between RAM and GPU in that case. However, when looking at the distribution of execution time for the deformation step only, shown in Fig. 9, we can see that making CUDA API calls incurs a relatively large overhead. The overhead remains constant for each kernel launch and memory transfer – regardless of the amount of data—with the memory transfers being responsible for most of that overhead.

### Comparison with other GPU-based methods

**Table 1:** Summary of method and reported performance for other GPU-based surgery simulators, compared to our method

**Paper**	**Solver type**	**Cutting**	**1000’s of nodes**	**Update rate**	**Method**
Pan [13]	Explicit	Yes	4.9	43	Meshless
Bosman [16]	Implicit	No	12.7	31	Meshless
Pietroni [17]	Implicit	Yes	0.6	15	Meshless
Courtecuisse [18]	Implicit	Yes	0.7	45	FEM
Hou [19]	Implicit	Yes	13	31	FEM
Camara [20]	Gauss-Seidel	No	5.5	79	PBD
Pan [21]	Gauss-Seidel	Yes	1.5	50	PBD
Dick [22]	Multigrid	No	38	62	FEM
Fenz [23]	Static	No	11	20	FEM
Our method	Implicit	Yes	25	60	Meshless

Table 1 presents an informal comparison of our method with other GPU-based surgery simulators. We achieve a large overall improvement in reported performance among all methods that allow cutting operations that we found in the literature. However, a direct comparison is made difficult by the fact that other reported results come from experiments run on older hardware. Furthermore, every other simulator that we found in the literature implements a different set of features, sometimes with more restrictions on cutting than our technique [19], sometimes with additionnal capabilities, such as a stable contact resolution method [18] or a heat transfer simulation as a criterion for cutting [13].

### Limitations

One obvious limitation of our data structure is that an element may not be connected to more nodes than the maximum allocated. While it has not occurred in any of our test cases, the possibility cannot be ruled out. Allocating more space would mean that a large portion of the structure would simply be empty. A better solution would be to guarantee that a situation requiring more than the maximum number of neighboring nodes can never occur. An approach that could achieve this would be to carefully choose the placement of nodes so that they cannot be coplanar.

Another potential issue is that the use of single precision arithmetic might result in a difference in the elastic behavior of simulated objects. This would be of particular interest when simulating a large number of elements, which could generate large position values with very small deformations, and very small corrections during individual CG iterations. More specific tests need to be performed to determine whether that is the case.

## Conclusion and future work

We have presented a data structure that can be used to describe graph structures in a way that can be read efficiently on graphics hardware, that is modified on the CPU and efficiently updated on the GPU with as little transferred data as possible. Our results show that this structure allows cutting objects simulated with a large number of elements at a sustained high update rate.

Future work will focus on integrating this method into an existing surgery simulator that enables a user to interact with virtual objects through a haptic device and provides highly detailed 3D visual feedback.

## Data Availability

The torus dataset model used in this study is available at “https://www.polymtl.ca/rv/torus.txt”. The datasets generated during the current study are available from the corresponding author on reasonable request.

## Abbreviations

API: Application programming interface
CPU: multi-core processors
EFG: Element Free Galerkin
FEM: Finite element methods
GPGPU: GPU computing
GPU: graphics processors
MSS: mass-spring system
PBD: Position-based dynamics
RAM: Random access memory
SIMT: Single instruction, multiple threads

## Authors’ information

Vincent Magnoux is a Ph.D. graduate from the department of Computer Engineering and Software Engineering at Polytechnique Montréal. His research focuses on real time physically based simulation in virtual reality, with a particular interest in high performance and parallel computing. Benoît Ozell is an associate professor in the department of Computer Engineering and Software Engineering at Polytechnique Montréal. His research interests include Computer Graphics, Scientific Visualization, Virtual Reality, Augmented Reality, Healthcare Simulations. https://www.polymtl.ca/rv/

## References

[1] Bianchi G, Harders M, Székely G. Mesh topology identification for mass-spring models. In: International Conference on Medical Image Computing and Computer-Assisted Intervention. Springer; 2003. p. 50–8.

[2] Cotin S, Delingette H, Ayache N (2000). A hybrid elastic model for real-time cutting, deformations, and force feedback for surgery training and simulation. Visual Computer..

[3] Belytschko T, Lu YY, Gu L (1994). Element-free Galerkin methods. Int J Numer Methods Eng.

[4] Müller M, Heidelberger B, Hennix M, Ratcliff J (2007). Position based dynamics. J Visual Commun Image Represent.

[5] Lindholm E, Nickolls J, Oberman S, Montrym J (2008). NVIDIA Tesla: A Unified Graphics and Computing Architecture. IEEE Micro..

[6] Georgii J, Westermann R (2005). Mass-spring systems on the GPU. Simul Modelling Practice Theory.

[7] Wu W, Heng PA (2004). A hybrid condensed finite element model with GPU acceleration for interactive 3D soft tissue cutting. Computer Anim Virtual Worlds..

[8] Yuan ZY, Ding YH, Zhang YY, Zhao JH (2010). Real-time simulation of tissue cutting with CUDA based on GPGPU. Adv Mater Res.

[9] Zerbato D, Baschirotto D, Baschirotto D, Botturi D, Fiorini P (2011). GPU-based physical cut in interactive haptic simulations. Int J Computer Assisted Radiol Surg.

[10] Comas O, Taylor ZA, Allard J, Ourselin S, Cotin S, Passenger J, Simulation Biomedical (2008). Efficient Nonlinear FEM for Soft Tissue Modelling and Its GPU Implementation within the Open Source Framework SOFA. Simulation biomedical.

[11] Taylor ZA, Comas O, Cheng M, Passenger J, Hawkes DJ, Atkinson D, et al. Modelling anisotropic viscoelasticity for real-time soft tissue simulation. In: International Conference on Medical Image Computing and Computer-Assisted Intervention. Springer; 2008. p. 703–710. 10.1007/978-3-540-85988-8_84 18979808

[12] Yibo S, Hui X, Dehai Y. Improvements of GPU Implementation of Nonlinear Soft Tissue Deformation with CHAI 3D. In: 3rd International Conference on Multimedia Technology (ICMT-13). Atlantis Press; 2013. p. 1196–1203.

[13] Pan J, Yang Y, Gao Y, Qin H, Si Y (2019). Real-time simulation of electrocautery procedure using meshfree methods in laparoscopic cholecystectomy. Visual Computer..

[14] Lapeer RJ, Gasson PD, Karri V (2011). A Hyperelastic Finite-Element Model of Human Skin for Interactive Real-Time Surgical Simulation. IEEE Trans Biomed Eng.

[15] Li S, Zhao Q, Wang S, Hao A, Qin H (2014). Interactive deformation and cutting simulation directly using patient-specific volumetric images. Computer Anim Virtual Worlds..

[16] Bosman J, Duriez C, Cotin S. Connective tissues simulation on GPU. In: VRIPHYS 13: 10th Workshop on Virtual Reality Interaction and Physical Simulation. Eurographics Association; 2013. p. 41–50.

[17] Pietroni N, Ganovelli F, Cignoni P, Scopigno R (2009). Splitting cubes: a fast and robust technique for virtual cutting. Visual Computer..

[18] Courtecuisse H, Jung H, Allard J, Duriez C, Lee DY, Cotin S (2010). GPU-based real-time soft tissue deformation with cutting and haptic feedback. Progr Biophys Mol Biol..

[19] Hou W, Liu PX, Zheng M (2019). A new model of soft tissue with constraints for interactive surgical simulation. Computer Methods Progr Biomed.

[20] Camara M, Mayer E, Darzi A, Pratt P (2016). Soft tissue deformation for surgical simulation: a position-based dynamics approach. Int J Computer Assisted Radiol Surg.

[21] Pan J, Bai J, Zhao X, Hao A, Qin H (2015). Real-time haptic manipulation and cutting of hybrid soft tissue models by extended position-based dynamics. Computer Animation Virtual Worlds..

[22] Dick C, Georgii J, Westermann R (2011). A real-time multigrid finite hexahedra method for elasticity simulation using CUDA. Simul Modelling Practice Theory..

[23] Fenz W, Dirnberger J. Real-time surgery simulation of intracranial aneurysm clipping with patient-specific geometries and haptic feedback. In: SPIE Medical Imaging. vol. 9415. International Society for Optics and Photonics; 2015. p. 94150H–94150H–10.

[24] Joldes GR, Wittek A, Miller K (2011). An adaptive dynamic relaxation method for solving nonlinear finite element problems. Application to brain shift estimation. Int J Numer Methods Biomed Eng..

[25] Baraff D, Witkin A. Large steps in cloth simulation. In: Proceedings of the 25th annual conference on Computer graphics and interactive techniques. ACM; 1998. p. 43–54.

[26] Magnoux V, Ozell B. Real-time visual and physical cutting of a meshless model deformed on a background grid. Computer Animation and Virtual Worlds. 2020; p. e1929.

[27] Magnoux V, Ozell B. Dynamic Cutting of a Meshless Model for Interactive Surgery Simulation (in press). In: Salento AVR 2020: 7th International Conference on Augmented Reality, Virtual Reality and Computer Graphics; 2020.

